# Gender-Specific Associations of Marine n-3 Fatty Acids and Fish Consumption with 10-Year Incidence of Stroke

**DOI:** 10.1371/journal.pone.0033866

**Published:** 2012-04-09

**Authors:** Janette de Goede, W. M. Monique Verschuren, Jolanda M. A. Boer, Daan Kromhout, Johanna M. Geleijnse

**Affiliations:** 1 Division of Human Nutrition, Wageningen University, Wageningen, The Netherlands; 2 National Institute for Public Health and the Environment, Bilthoven, The Netherlands; University of Swansea, United Kingdom

## Abstract

**Background:**

There is some evidence that the association of fish and marine fatty acids with stroke risk differs between men and women. We investigated the gender-specific associations of habitual intake of the marine fatty acids eicosapentaenoic acid (EPA) plus docosahexaenoic acid (DHA) and fish on incident stroke in a population-based study in the Netherlands.

**Methods:**

We prospectively followed 20,069 men and women, aged 20–65 years, without cardiovascular diseases at baseline. Habitual diet was assessed with a validated 178-item food frequency questionnaire. Incidence of stroke was assessed through linkage with mortality and morbidity registers. Cox proportional hazards models were used to estimate multivariable-adjusted hazard ratios (HR) and 95% confidence intervals (95%CI).

**Results:**

During 8–13 years of follow-up, 221 strokes occurred. In women, an inverse dose-response relation (P-trend = 0.02) was observed between EPA-DHA intake and incident stroke, with an HR of 0.49 (95% CI: 0.27–0.91) in the top quartile of EPA-DHA (median 225 mg/d) as compared to the bottom quartile (median 36 mg/d). In men, the HR (95%CI) for the top quartile of EPA-DHA intake was 0.87 (0.51–1.48) (P-trend = 0.36). Similar results were observed for fish consumption and stroke incidence.

**Conclusion:**

A higher EPA-DHA and fish intake is related to a lower stroke risk in women, while for men an inverse association could not be demonstrated.

## Introduction

Worldwide, stroke is the second largest cause of mortality and a major cause of long-term disability [1], [2]. As part of a healthy diet, fish consumption is advised to reduce the risk of cardiovascular diseases [3], [4], [5]. Although literature strongly suggests that consuming fish protects against coronary heart disease [6], data for protection against stroke are less convincing.

Several [7], [8], [9], [10], [11], [12], [13], although not all [14], [15], [16], [17], [18], prospective cohort studies showed inverse associations of fish consumption with stroke. In a meta-analysis, He *et al* summarized prospective cohort studies published through 2003 and concluded that fish consumption once a week compared to less than once per month was related to a 13% (HR:0.87; 95%CI: 0.77–0.98) lower stroke risk [19]. In three cohort studies [9], [10], [11] with information on types of stroke, consuming fish more than once a month was associated with a 30–35% lower risk of ischemic stroke, and not to hemorrhagic stroke [19].

In four [8], [9], [12], [13] out of six [8], [9], [12], [13], [17], [20] prospective studies carried out in women from western countries, fish consumption was inversely associated with stroke, whereas only two [7], [10] out of seven [7], [8], [10], [12], [14], [15], [20] studies reported inverse associations in men. In addition, fish intake was more strongly inversely related to stroke in women than in men in two cohort studies that stratified by gender [8], [12]. Less data are available for the intake of the fatty acids eicosapentaenoic acid (EPA) and docosahexaenoic acid (DHA) from fish, but those available were in agreement with those on fish consumption and stroke incidence [9], [10], [14], [16], [18].

We investigated the gender-specific associations of habitual intake of EPA-DHA and fish with 10-year incidence of stroke in a large population-based study in the Netherlands.

## Methods

### Ethical Statement

This research was performed in accordance with the ethical principles for medical research involving human subjects outlined in the Declaration of Helsinki. This research was approved by the Medical Ethics Committee of TNO Prevention and Health (Leiden, The Netherlands). All participants gave written informed consent.

### Design and study population

The “Monitoring Project on Chronic Disease Risk Factors” (MORGEN Study) is a population-based cohort of 22,654 men and women, aged 20–65 years in the Netherlands. The MORGEN Study contributes to the Dutch part of the European Prospective Investigation into Cancer and Nutrition (EPIC) [21]. Baseline (1993–1997) information on diet, lifestyle, and cardiovascular risk factors was collected and participants were followed for cardiovascular endpoints. The study complies with the Declaration of Helsinki and the protocol was approved by the Medical Ethics Committee of the TNO Prevention and Health Institute (Leiden, The Netherlands). Written informed consent was obtained from each participant.

Participants who did not provide informed consent for vital status follow-up were excluded (n = 701) as well as participants with no dietary information (n = 72) or with extreme energy intakes (<2,094 or >18,844 kJ for women and <3,350 or >20,938 kJ for men; n = 97). Furthermore, we excluded 442 participants with a history of myocardial infarction or stroke at baseline. We additionally excluded participants with self-reported diabetes and participants who used serum lipid modifying agents or antihypertensive drugs, resulting in 20,069 participants (8,988 men and 11,081 women) for the present analysis.

### Dietary assessment

Habitual diet was assessed at baseline with a self-administered 178-item Food Frequency Questionnaire (FFQ), covering the previous year [22], [23]. Participants indicated consumption of main food groups in times per day, per week, per month, or as never, combined with questions on the relative intake of foods within food groups (seldom/never, sometimes, often, mostly/always). Information on habitual fish intake was obtained by questions on the absolute frequency of fish consumption combined with questions on the following types of fish: 1. lean and moderately fatty fish, including plaice, cod, fried fish, fish fingers; 2. fatty fish, including eel, mackerel, herring; 3. shrimps and mussels. Nutrient intakes were calculated with the “Dutch food composition database” of 1998. For individual fatty acids, we used the updated table of 2001.

The relative validity of the FFQ against 12 monthly 24-h recalls and reproducibility of the FFQ after 6 months for food groups and nutrients were assessed among 121 Dutch men and women [22], [23]. The Spearman rank correlations for the reproducibility of the FFQ after 6 months for fish intake were 0.49 for men and 0.61 for women. The relative validity (Spearman correlation) of the FFQ for fish intake was 0.32 for men and 0.37 for women [23]. The rank correlations for dietary EPA and DHA (mg/d) with cholesteryl ester plasma levels in a subset of the current population were in men (n = 268) 0.34 for EPA and 0.47 for DHA and in women (n = 189) 0.36 for EPA and 0.33 for DHA (unpublished results).

### Case ascertainment and follow-up

Vital status was checked through linkage with the municipal population registers. For those who died, information on the cause of death was obtained from Statistics Netherlands. Information on nonfatal stroke was provided by the national hospital discharge register based on a validated probabilistic method described in more detail elsewhere [24]. On the national level, data from the Dutch hospital discharge register can be uniquely matched to a single person for at least 88% of the hospital admissions [24]. Incident total stroke comprised fatal and nonfatal stroke, corresponding with International Classification of Diseases (ICD-10, WHO) codes I60–I66 and G45. This definition also included transient ischemic attacks (TIA) (G45). Ischemic stroke included I63, I65, I66, and G45, and hemorrhagic stroke included I60–I62. For hospital admissions and for causes of death coded until January 1, 1996, corresponding ICD9 codes were used. If the dates of hospital admission and death coincided, the event was considered fatal.

### Data collection on risk factors

The baseline measurements were previously described in detail by Verschuren *et al* [25]. Body weight, height, and blood pressure were measured by trained research nurses. Blood pressure was measured twice, with the subject in sitting position. The mean of the two measurements was used in the analyses. Non-fasting blood was analyzed for plasma total and high-density lipoprotein (HDL) cholesterol. A self-administered questionnaire was used to assess the presence of diabetes, history of myocardial infarction or stroke, medication use, parental history of premature myocardial infarction, education level, and cigarette smoking. Alcohol intake (based on the FFQ) was calculated in glasses/d and was categorized as no intake, low to moderate intake (men ≤2 and women ≤1 glasses/d), or high intake (men>2 and women >1 glasses/d). Baseline physical activity was assessed with a validated questionnaire in 76% of the cohort, enrolled between 1994–1997 [26]. For this subset, we calculated whether participants were engaged in cycling (yes/no) and sports (yes/no), both activities with a metabolic equivalent score ≥4, which were significantly inversely related to cardiovascular disease incidence in this population [27].

### Statistical analysis

Follow-up time was calculated from date of enrollment until death, incident stroke, date of loss-to-follow-up due to emigration out of the Netherlands (n = 693) or 1 January 2006, whichever occurred first. We used Cox proportional hazard models to estimate hazard ratios (HR) with 95% confidence intervals (95% CI) for the association of gender-specific quartiles of EPA-DHA and total fish intake with stroke incidence. The analyses were repeated for stroke subtypes, i.e. ischemic stroke and hemorrhagic stroke. The proportional hazards assumption was tested and not rejected based on Schoenfeld residuals and visual inspection. Participants' characteristics in quartiles of EPA-DHA intake are presented as mean±SD, median [interquartile range (Q1–Q3)], or percentages, depending on the type and distribution of variables. Interactions of EPA-DHA and fish intake with gender were statistically tested with the likelihood ratio test comparing the fully adjusted model of the total group (men and women combined) with a similar model with additional product terms of gender and quartiles of intake. The correlation between the intake of EPA-DHA and total fish was assessed with the Spearman rank correlation test.

In addition to an age adjusted model [model 1], we used multivariable-adjusted models [model 2] that included total energy intake (kcal/d), body mass index (kg/m^2^), alcohol intake (none, low to moderate, or high), cigarette smoking (never, former, current), educational level (primary school, secondary school, up to higher vocational training, completed higher vocational training or university), parental history of premature myocardial infarction (yes/no; for father <55 y and for mother <65 y), intake of dietary fiber (g/d), vitamin C (mg/d), beta-carotene (mg/d), saturated fatty acids (en%), trans fatty acids (en%), monounsaturated fatty acids (en%), linoleic acid (en%), and alpha-linolenic acid (en%).

To examine whether systolic blood pressure could be an intermediate factor in the association of EPA-DHA or fish intake with stroke, we added this variable to the multivariable model and examined changes in HRs. Possible confounding by physical activity (cycling and sports) was checked in the subgroup of participants for which these data were available (n = 15,423). All p-values are two-tailed with α = 0.05. Statistical analyses were performed with Statistical Analysis Software (SAS), version 9.2.

## Results

The median EPA-DHA intake across quartiles varied from 36 to 225 mg/d in women and from 44 to 241 mg/d in men. In the higher quartiles participants were slightly older, higher educated, and they consumed more energy and alcohol. In men, but not in women, EPA-DHA intake was positively associated with current smoking (**Table 1**). Eight per cent of the participants never consumed fish and 40% consumed fish less than once per month. The median fish consumption was twice per month. The Spearman rank correlation between total fish consumption and EPA-DHA intake was 0.95. We observed no interaction between EPA-DHA or fish intake and gender in relation to incident stroke (p for interaction = 0.30, both for EPA-DHA and fish).

**Table 1:** Baseline characteristics of 20,069 Dutch men and women, aged 20–65 y, by quartiles of EPA-DHA intake.

	Women				Men			
Range EPA-DHA (median), mg/d	Q1<57(36)	Q257–106(77)	Q3107–188 (142)	Q4>188(225)	Q1<66(44)	Q266–118(89)	Q3119–198(157)	Q4>198(241)
N	2770	2770	2771	2770	2247	2247	2247	2247
Age, y	40.5±11.9	40.3±11.1	41.1±10.8	42.5±10.8	41.2±11.4	41.2±11.0	42.2±10.6	43.2±10.7
Fish, g/d	1.3±1.3	4.7±2.1	10.6±3.5	22.0±14.2	1.5±1.4	5.0±2.2	11.1±3.8	21.9±13.9
EPA, mg/d	10±5	24±7	46±10	94±58	11±6	26±7	49±11	102±63
DHA, mg/d	26±10	56±11	99±18	189±103	31±12	65±12	109±18	202±105
ALA, en%	0.55±0.17	0.55±0.15	0.56±0.16	0.56±0.15	0.53±0.15	0.54±0.15	0.53±0.15	0.54±0.15
Linoleic acid, en%	5.26±1.60	5.36±1.47	5.43±1.49	5.53±1.58	5.28±1.60	5.39±1.55	5.36±1.56	5.46±1.63
Saturated fatty acids, en%	14.7±2.6	14.7±2.4	14.4±2.5	14.1±2.6	14.4±2.5	14.4±2.4	14.2±2.4	14.2±2.5
Total fatty acids, en%	34.9±5.2	35.5±4.9	35.1±5.0	34.9±5.1	34.6±5.0	35.1±4.7	34.7±5.0	35.0±5.0
Total energy, MJ/d	7.9±2.1	8.4±2.1	8.5±2.1	8.6±2.3	10.6±2.7	11.0±2.8	11.0±2.8	11.3±2.9
Body mass index, kg/m^2^	24.4±4.1	24.5±4.1	24.4±4.1	24.7±4.1	25.1±3.6	25.3±3.4	25.2±3.3	25.4±3.5
Smoking, %								
Never	39	36	35	36	34	31	31	28
Former	24	28	28	27	32	34	34	30
Current	37	36	37	37	34	35	35	42
Alcohol consumption, %								
None	23	16	15	16	10	7	7	8
Low to moderate	60	62	58	53	62	61	56	50
High	17	22	27	30	28	32	37	41
Highly educated,^1^ %	17	21	26	26	24	26	32	29
Dutch ethnicity, %	98	98	97	95	98	96	96	94
Physical activity,^2^ %								
Engaged in cycling	60	62	60	61	55	59	59	58
Engaged in sports	33	37	39	38	36	40	40	37
Parental history of premature MI, %	10	9	8	9	9	9	9	9
Serum total cholesterol,^3^ mmol/l	5.2±1.0	5.2±1.0	5.2±1.1	5.3±1.0	5.2±1.0	5.3±1.1	5.3±1.1	5.4±1.1
Serum HDL-cholesterol,^3^ mmol/l	1.5±0.4	1.5±0.4	1.5±0.4	1.5±0.4	1.2±0.3	1.2±0.3	1.2±0.3	1.2±0.3
Systolic blood pressure, mm Hg	116.7±15.5	116.2±15.6	116.2±14.9	117.1±15.8	123.7±13.9	124.1±14.6	123.9±14.6	124.4±15.5
Diastolic blood pressure, mm Hg	74.3±9.9	74.5±10.3	73.8±9.9	74.2±10.3	78.4±9.9	78.5±10.1	78.5±10.1	78.4±10.7

Abbreviations: EPA, eicosapentaenoic acid; DHA, docosahexaenoic acid; HDL, High Density Lipoprotein; Q, quartiles.

1 University or higher vocation training.

2 Available for participants enrolled between 1994 and 1997 (n = 15,423).

3 Nonfasting.

During 8–13 years of follow-up (median 10.5 years), 221 incident strokes occurred. Total stroke comprised 142 ischemic strokes (including 60 TIAs), 47 hemorrhagic strokes, and 32 unspecified strokes. Rates for TIA were similar for men and women. Men had higher incidence rates of total and ischemic stroke (excluding TIA), whereas women had higher rates of hemorrhagic stroke (**Table 2**). Women were on average 5 years younger (∼47 y) than men (∼52 y) when their first stroke occurred.

**Table 2:** Incidence rates of total stroke and stroke subtypes in 20,069 Dutch men and women, aged 20–65 y.

	Women		Men	
	Incidence rate^1^	%	Incidence rate	%
Total stroke^2^	9.2		12.4	
Ischemic stroke excluding TIA	2.6	28	5.6	45
TIA	2.9	31	2.9	24
Hemorrhagic stroke	2.7	29	1.7	14
Unspecified	1.0	11	2.2	17

Abbreviation: TIA, transient ischemic attack.

1 Incidence rates per 10,000 person years.

2 International Classification of Diseases (ICD-10) codes were I60–I66 and G45 for total stroke; I63, I65, I66, for ischemic stroke excluding TIA, G45 for TIA, and I60–I62 for hemorrhagic stroke.

After adjustment for potential confounders, we found an inverse dose-response relation (p-trend = 0.02) of EPA-DHA intake with incident total stroke in women. The HR (95%CI) for quartile 4 (median intake: 225 mg/d) was 0.49 (0.27–0.91) compared to the bottom quartile (median intake: 36 mg/d). EPA-DHA intake was not significantly associated with incident stroke in men. The HR for total stroke in quartile 4 was 0.87 (0.51–1.48) (**Table 3**). Results of fish consumption were similar to those of EPA-DHA intake. Women in the top quartile of fish consumption had a significantly lower risk of total stroke (HR: 0.49; 95%CI: 0.26–0.94) (p-trend = 0.01), while men in the top quartile had a HR of 0.75 (95%CI: 0.44–1.26) (**Table 4**).

**Table 3:** Associations of incident stroke by quartiles of EPA-DHA intake in 20,069 Dutch men and women^1^.

	Total stroke^2^		Ischemic stroke		Hemorrhagic stroke	
Intake, mg/d	Cases	Model 1^3^	Model 2^4^	Cases	Model 2^4^	Cases	Model 2^4^
Range (median)		HR (95% CI)	HR (95% CI)		HR (95% CI)		HR (95% CI)
Women							
Q1: <57 (36)	33	1.0 (ref)	1.0 (ref)	19	1.0 (ref)	9	1.0 (ref)
Q2: 57–106 (77)	28	0.88 (0.53–1.45)	0.89 (0.53–1.49)	17	0.98 (0.50–1.91)	7	0.73 (0.27–2.00)
Q3: 107–188 (142)	28	0.86 (0.52–1.42)	0.86 (0.51–1.46)	17	0.98 (0.50–1.93)	10	1.00 (0.39–2.57)
Q4: >188 (225)	17	0.49 (0.28–0.89)	0.49 (0.27–0.91)	11	0.62 (0.29–1.35)	5	0.45 (0.14–1.42)
*P trend*		*0.02*	*0.02*		*0.21*		*0.18*
Men							
Q1: <66 (44)	30	1.0 (ref)	1.0 (ref)	22	1.0 (ref)	6	1.0 (ref)
Q2: 66–118 (89)	33	1.13 (0.69–1.86)	1.16 (0.70–1.92)	20	0.93 (0.50–1.74)	7	1.22 (0.40–3.70)
Q3: 119–198 (157)	24	0.78 (0.46–1.34)	0.84 (0.48–1.45)	18	0.87 (0.46–1.65)	1	0.16 (0.02–1.32)
Q4: >199 (241)	28	0.86 (0.51–1.44)	0.87 (0.51–1.48)	20	0.85 (0.45–1.60)	2	0.28 (0.05–1.46)
*P trend*		*0.32*	*0.36*		*0.61*		*0.03*

Abbreviations: EPA, eicosapentaenoic acid; DHA, docosahexaenoic acid; HR, hazard ratio; CI, confidence interval.

1 Values are HR with 95% CI in quartiles (Q1–Q4) of EPA-DHA intake, using Q1 as the reference category.

2 International Classification of Diseases (ICD-10) codes were I60–I66 and G45 for total stroke; I63, I65, I66, and G45 for ischemic stroke and I60–I62 for hemorrhagic stroke.

3 Model 1: adjusted for age.

4 Model 3: additionally adjusted for smoking, BMI, educational level, parental history of myocardial infarction, alcohol intake, total energy intake, dietary fiber, vitamin C, beta-carotene, saturated fatty acids, trans fatty acids, monounsaturated fatty acids, linoleic acid, and alpha-linolenic acid.

**Table 4:** Associations of incident stroke by quartiles of fish consumption in 20,069 Dutch men and women^1^.

	Total stroke^2^			Ischemic stroke		Hemorrhagic stroke	
Intake, g/d	Cases	Model 1^3^	Model 2^4^	Cases	Model 2^4^	Cases	Model 2^4^
Range (median)		HR (95% CI)	HR (95%CI)		HR 95%CI		HR 95% CI
Women							
Q1 (n = 2764): <3.0 (1.0)	29	1.0 (ref)	1.0 (ref)	17	1.0 (ref)	6	1.0 (ref)
Q2 (n = 2776): 3.0–7.2 (4.2)	34	1.21 (0.74–1.98)	1.25 (0.75–2.08)	20	1.25 (0.65–2.41)	12	1.97 (0.73–5.31)
Q3 (n = 2757): 7.3–14.0 (9.8)	28	0.96 (0.57–1.61)	1.00 (0.59–1.71)	18	1.14 (0.58–2.24)	8	1.19 (0.41–3.52)
Q4 (n = 2785): >14.0 (18.0)	15	0.48 (0.26–0.90)	0.49 (0.26–0.94)	9	0.54 (0.24–1.23)	5	0.67 (0.19–2.29)
*P trend*		*0.01*	*0.01*		*0.10*		*0.19*
Men							
Q1 (n = 2249): <3.3 (1.1)	32	1.0 (ref)	1.0 (ref)	22	1.0 (ref)	5	1.0 (ref)
Q2 (n = 2216): 3.3–7.4 (4.3)	32	1.03 (0.63–1.68)	1.04 (0.63–1.72)	22	1.05 (0.57–1.93)	7	1.52 (0.48–4.85)
Q3 (n = 2300): 7.5–14.0 (10.8)	24	0.68 (0.40–1.16)	0.73 (0.42–1.24)	17	0.77 (0.40–1.47)	3	0.57 (0.13–2.44)
Q4 (n = 2223): >14.0 (17.6)	27	0.74 (0.44–1.23)	0.75 (0.44–1.26)	19	0.79 (0.42–1.48)	1	0.17 (0.02–1.50)
*P trend*		*0.11*	*0.13*		*0.27*		*0.04*

Abbreviations: HR: hazard ratio, CI: confidence interval.

1 Values are HR with 95% CI in quartiles (Q1–Q4) of fish intake, using Q1 as the reference category.

2 International Classification of Diseases (ICD-10) codes were I60–I66 and G45 for total stroke; I63, I65, I66, and G45 for ischemic stroke and I60–I62 for hemorrhagic stroke.

3 Model 1: adjusted for age.

4 Model 2: additionally adjusted for smoking, BMI, educational level, parental history of myocardial infarction, alcohol intake, total energy intake, dietary fiber, vitamin C, beta-carotene, saturated fatty acids, trans fatty acids, monounsaturated fatty acids, linoleic acid, and alpha-linolenic acid.

The associations of EPA-DHA and fish consumption with ischemic stroke and total stroke were similar. The HR (95%CI) for ischemic stroke risk in the top quartile of EPA-DHA intake was 0.62 (0.29–1.35) for women and 0.85 (0.45–1.60) for men. However, confidence intervals were wider compared to total stroke and the associations were not significant in either men or women. Results for total and ischemic stroke with or without TIA were also similar. The HR (95% CI) for total stroke without TIA in quartiles 2–4 of EPA-DHA were 0.78 (0.42–1.43), 0.80 (0.43–1.48), and 0.44 (0.21–0.90) in women and 1.16 (0.65–2.08), 0.91 (0.50–1.69), and 0.91 (0.50–1.66) in men. Although we had a limited number of cases, the associations of EPA-DHA and fish with hemorrhagic stroke suggested also an inverse association.

Additional adjustment for systolic blood pressure did not change the associations. HRs (95%CI) for total stroke incidence in the top quartile of EPA-DHA were 0.50 (0.27–0.92) for women and 0.85 (0.50–1.46) for men. In the top quartile of fish consumption, HRs for total stroke were 0.50 (0.26–0.95) and 0.74 (0.44–1.25) and for men and women, respectively. For the subgroup with information on physical activity (n = 15,423), the full model with and without physical activity yielded similar results (results not shown).

## Discussion

In this prospective cohort study from the Netherlands, a higher EPA-DHA and fish intake were associated with a lower stroke risk in women, with no differences between stroke types. For men, these associations were weaker and not statistically significant.

This study has several strengths, including complete information on vital status with little loss to follow up of a large population-based cohort and detailed information on potential confounders. An extensive FFQ was used that allowed calculation of EPA+DHA from the whole diet. However, there were also limitations. Misclassification of participants for fish or EPA-DHA intake may have occurred. However, correlations for EPA and DHA intake derived from the FFQ with levels in plasma cholesteryl esters were 0.32 and 0.41, which is comparable to other studies [28]. Furthermore, we excluded participants with a history of myocardial infarction or stroke and participants on cholesterol or blood pressure lowering medication, because those participants may have changed their diets. We therefore consider potential misclassification at baseline random rather than dependent on disease outcome.

Nonfatal stroke events were assessed through probabilistic linkage with the national hospital discharge register. If we have missed events by this procedure, this is unlikely to be related to EPA-DHA or fish intake, and will therefore not have biased our results. In the Netherlands, brain imaging is used to identify stroke in 98% of the admitted stroke patients [29]. In contrast to most other studies on stroke, we also evaluated TIA (which comprised 42% of ischemic stroke cases), a less severe stroke event of which symptoms last <24 h. Because the results for incident stroke with or without TIA were similar in our study, we included TIA to increase statistical power.

We observed a significant 51% lower risk of total stroke in women in the highest quartile of EPA-DHA (>188 mg/d), or fish (>14 g/d) intake. The difference with the bottom quartile corresponded to ∼one portion of fish per week. Our associations for EPA-DHA were stronger than in the Nurses' Health Study. In that study, EPA-DHA intakes in quintile 3 (median 171 mg/d) and 4 (median 221 mg/d), which approximately represent our top quartile, were associated with a respectively 31% and 17% lower stroke risk, compared to the bottom quintile [9]. American cohort studies reported a 23% (HR: 0.77; 95%CI: 0.53–1.13) [8] and a 22% lower (HR: 0.78; 95% CI: 0.55–1.12) [9] stroke risk for women who consumed one fish meal per week compared to no fish [8] or less than once per month [9]. For women from the UK [12] and Sweden [12], [13], eating fish once or twice per week compared to less than once per week was associated with borderline significant lower stroke incidences of 26% (UK) and 13% (Sweden). In summary, cohort studies from Western countries have consistently shown that EPA-DHA and fish intake are inversely associated with stroke risk in women, with HRs varying between 0.5 and 0.8.

We found no significant association for EPA-DHA with stroke in men. In the Health Professionals Follow up Study, the largest male cohort, a 23% lower stroke risk (HR: 0.77; 95% CI: 0.52–1.14) was observed for an EPA-DHA intake of 200–400 mg/d vs. less than 50 mg/d after 12 years of follow-up [10]. In the Physicians' Health Study, however, EPA-DHA intake was not associated with 4-year incidence of stroke [14]. Although not significant, the HR of 0.75 for stroke in the top vs. bottom quartile of fish consumption in our male participants was in line with the borderline significant 26% lower stroke risk in the American health professionals consuming one fish meal per week compared to less than one per month [10]. Our stroke risk estimate of 0.75 was stronger than the male-specific estimate of 0.90 (95%CI: 0.78–1.04) for fish once per week vs. less than once per month from the meta-analysis of He *et al* [19]. That effect size estimate, however, was diluted by a large Chinese study that reported a positive association between fish intake and fatal stroke [16]. In men from the UK, eating fish (mainly processed and fried) once or twice per week compared to less than once per week was associated with a non-significant higher stroke risk [12]. To summarize, in cohort studies from Western countries with a similar range of intake compared to our study, inverse associations for men are less convincing than for women.

Concluding, evidence is accumulating that a higher EPA-DHA and fish intake is related to a lower stroke risk in women, whereas for men an inverse association could not be demonstrated. This gender difference cannot be explained by differences in stroke types as inverse associations were observed both for ischemic and hemorrhagic stroke. Furthermore, distributions of EPA-DHA and fish intake for men and women were similar. If this gender difference will be confirmed in other and larger studies, research is needed to clarify the physiologic difference of this epidemiologic finding.
